# The Diverse Experiences and Challenges of Individuals With Alopecia Areata: Survey Results From Finland

**DOI:** 10.1177/23743735251346633

**Published:** 2025-06-03

**Authors:** Johanna M. Mandelin, Laura Rehn, Mikko Kosunen, Merja Väkeväinen, Saara Rannanpää

**Affiliations:** 1 Department of Dermatology and Allergology, 3835 University of Helsinki and Helsinki University Hospital, Helsinki, Finland; 2 Helsinki City Hospital, Helsinki, Finland; 3 Mehiläinen, Helsinki, Finland; 4 Pfizer, Helsinki, Finland

**Keywords:** alopecia areata, comorbidity, hair loss, inflammation, patient experience, wig

## Abstract

Alopecia areata (AA) is a chronic disease that significantly impacts well-being and quality of life. Despite its burden, professional support and treatment utilization remain low. This study investigated the experiences of 226 individuals in Finland with AA through an online survey. Most (82.7%) had at least one comorbid condition, most commonly atopic dermatitis (45.1%) and allergic rhinitis (43.4%). Depression and anxiety were reported by 22.1% and 23.0% of respondents, respectively. Those with ≥50% hair loss were significantly less likely to report higher overall well-being (odds ratio 0.60; 95% CI 0.38-0.96; *P* = .03), and experienced a greater negative impact on their relationships and social situations compared to those with <50% hair loss. The majority (82.7%) had not received any treatment for AA during the past year. Social media and peer support were the most common information sources. These findings highlight the need for improved patient education, and professional support. Healthcare providers should proactively offer guidance on treatment options and assess comorbidities to improve individual well-being.

## Plain Language Summary

This online survey looked at how individuals with alopecia areata (AA) experience their condition and its treatment in Finland. Alopecia areata is a chronic autoimmune condition that causes hair loss, often in patches, and can affect both the scalp and other areas of the body. It is not just a cosmetic issue but a disease that has far-reaching physical, emotional, and psychological impacts. Out of 226 respondents who took part in the survey, many reported feeling a significant impact on their well-being, including their self-esteem and social interactions. Most respondents had other health conditions, such as atopic dermatitis and allergies, and many also reported symptoms of anxiety or depression. Those with more severe hair loss (50% or more) were more likely to struggle with their overall well-being, relationships, and social situations. Additionally, most respondents had not received any treatment for their condition recently, and many were unhappy with their current treatment options. Financial burdens, like out-of-pocket costs for wigs, also added stress. The survey shows that individuals with AA need better treatment options and more reliable information. Many rely on social media and peer support for help, which suggests a gap in the support they receive from healthcare professionals. Overall, the findings highlight the need for more comprehensive care and support for both the physical and emotional challenges of AA.

## Introduction

Alopecia areata (AA) is an autoimmune disease characterized by nonscarring hair loss, ranging from small bald patches to complete loss of hair on the scalp, face, and/or body.^1^ The extent of hair loss varies, from patchy hair loss (AA) to complete scalp hair loss (AT, alopecia totalis) or total loss of body hair (AU, alopecia universalis).^1^ In this article, AA refers to all these forms of the disease. Alopecia areata affects approximately 2% of the global population^2^ and occurs in both children and adults, with peak incidence in young adulthood.^3^

Since AA is a chronic disease with an uncertain prognosis, it frequently causes stress and anxiety for those affected.^1,4^ Beyond its cosmetic impact, AA is associated with a higher burden of autoimmune, inflammatory, and mental health conditions, significantly diminishing the quality of life across multiple aspects.^5–8^ An earlier study from Northern Finland revealed that over 60% of individuals with AA had one or more comorbid conditions in their medical records. Atopic dermatitis was the most common condition, reported by 1 in 4 individuals with AA.^9^ In addition, several studies have shown that anxiety, depression, sleep disturbances, and suicides are more prevalent in individuals with AA than in the general population.^4,10,11^

In addition to the physical and mental health burden of AA, out-of-pocket costs can impose a significant financial strain on individuals, often surpassing those associated with other chronic diseases. Expenses for items such as wigs and pigmentation treatments further exacerbate this burden and should be taken into account in economic evaluations of the disease's overall impact.^12,13^

Over the years, there has been increased understanding of the pathogenesis, diagnostics, and treatment approaches for AA. Treatment options for AA vary widely, ranging from nonmedical approaches, such as opting for no treatment, microblading, or the use of hair prosthetics, to medical interventions like the immuno-modulating Janus kinase (JAK) inhibitors.^4^ Despite this progress, challenges in managing and reducing the burden of the disease persist. The aim of this survey study was to examine the impact of AA on various aspects of life, with a specific emphasis on comorbidities, well-being, financial burdens, and treatment satisfaction among individuals with AA in Finland. To our knowledge, self-reported data on these aspects have not been comprehensively reported earlier from Finland. Moreover, the findings can provide valuable insights not only into understanding the needs of people with AA but also for those with other skin conditions that share similar psychosocial and healthcare challenges, both within and beyond Finland. The advantage of surveys over healthcare registry data lies in their ability to capture the holistic impact of the disease on individuals.

## Methods

The online survey questionnaire was developed in collaboration with a patient organization, The Finnish Allergy, Skin and Asthma Federation. The respondents were recruited through the organization's website and social media channels. Anonymous data were securely collected on a comprehensive software solution (Questback) between May 11, 2023, and June 20, 2023. All participants consented for the use of their data at aggregated level before proceeding with the survey. According to Finnish law, this type of survey study does not require ethical approval.

All data were self-reported by the respondents. It was also possible to complete the survey on behalf of a child or with their consent. The survey included a total of 42 quantitative and qualitative questions related to disease-specific variables, such as diagnosis, treatments, comorbidities, associated costs, and the impact of the disease on everyday life. The analysis was limited to respondents who reported their disease subtype as AA, AT, or AU. Other respondents were excluded (eg, alopecia androgenica). For comparison, respondents were divided into 2 groups based on the self-reported extent of hair loss: less than or at least 50% hair loss. Respondents only reported their percentage of hair loss and were not aware of the 50% cutoff level.

The quantitative data were analyzed with IBM SPSS Version 28 statistical software package. Associations between extent of hair loss and ordinal survey responses were analyzed by ordinal regression using the “OrderedModel” function of the “statsmodels” Python library (vers. 0.13.5). Results were odds ratio (OR) with 95% confidence intervals, and associated *P* values. Associations for continuous variable survey responses were analyzed by linear regression using the ordinary least squares function of the “statsmodel” library, with results presented as linear regression coefficients (β) with 95% confidence intervals, and associated *P* values.

## Results

### Demographics and Comorbidities

A total of 226 eligible respondents completed the survey (Table 1). The respondents were predominantly women (93.8%), and represented all age groups, with the majority (45.6%) being middle-aged (40-59 years old). Many had received their diagnosis during childhood or adolescence (38.1%), and within 2 months from the first contact to health care (60.6%). The majority reported having a patchy AA (70.8%), while the remaining had either AT (8.4%) or AU (20.8%). Alopecia areata was mostly diagnosed in public healthcare: 48.3% in primary care and 34.5% in specialized care. About half (51.8%) of the respondents reported to be members of a patient organization.

**Table 1.** Demographic Characteristics of the Study Population.

Characteristics	Survey respondents, n (%)
	N = 226
Sex	
Female	212 (93.8)
Male	8 (3.5)
Age groups	
<18	16 (7.1)
18-29	21 (9.3)
30-39	39 (17.3)
40-49	52 (23.0)
50-59	51 (22.6)
≥60	47 (20.8)
Age at diagnosis	
<18	86 (38.1)
18-29	55 (24.3)
30-39	32 (14.2)
40-49	21 (9.3)
50-59	25 (11.1)
≥60	7 (3.1)
Time from diagnosis	
<1 year	11 (4.9)
1-2 years	20 (8.8)
3-4 years	29 (12.8)
>5 years	160 (70.8)
Not sure / do not remember	6 (2.7)
Time from first symptoms to healthcare contact	
<1 month	58 (25.7)
1-2 months	53 (23.5)
3-5 months	34 (15.0)
6-11 months	10 (4.4)
1-2 years	16 (7.1)
>3 years	14 (6.2)
Not sure / do not remember	41 (18.1)
Time to diagnosis after contacting healthcare	
<1 month	95 (42.0)
1-2 months	42 (18.6)
3-5 months	18 (8.0)
6-11 months	9 (4.0)
>1 year	7 (3.1)
Not sure / do not remember	55 (24.3)
Healthcare unit where the diagnosis was made	
Primary healthcare	109 (48.3)
Specialized healthcare, university hospital	78 (34.5)
Private clinic	39 (17.3)
Type of alopecia	
Alopecia areata (AA)	160 (70.8)
Alopecia totalis (AT)	19 (8.4)
Alopecia universalis (AU)	47 (20.8)
Percentage of hair loss	
<50	105 (46.5)
50-90	37 (16.4)
>90	82 (36.4)
Education	
Comprehensive school	16 (7.1)
Upper secondary	96 (42.5)
Higher secondary	106 (46.9)
Other	5 (2.2)
Employment	
Full time	141 (62.4)
Part time	16 (7.1)
Student	32 (14.2)
Unemployed	6 (2.7)
Retired	23 (10.2)
Other	8 (3.5)

**Figure 1.**
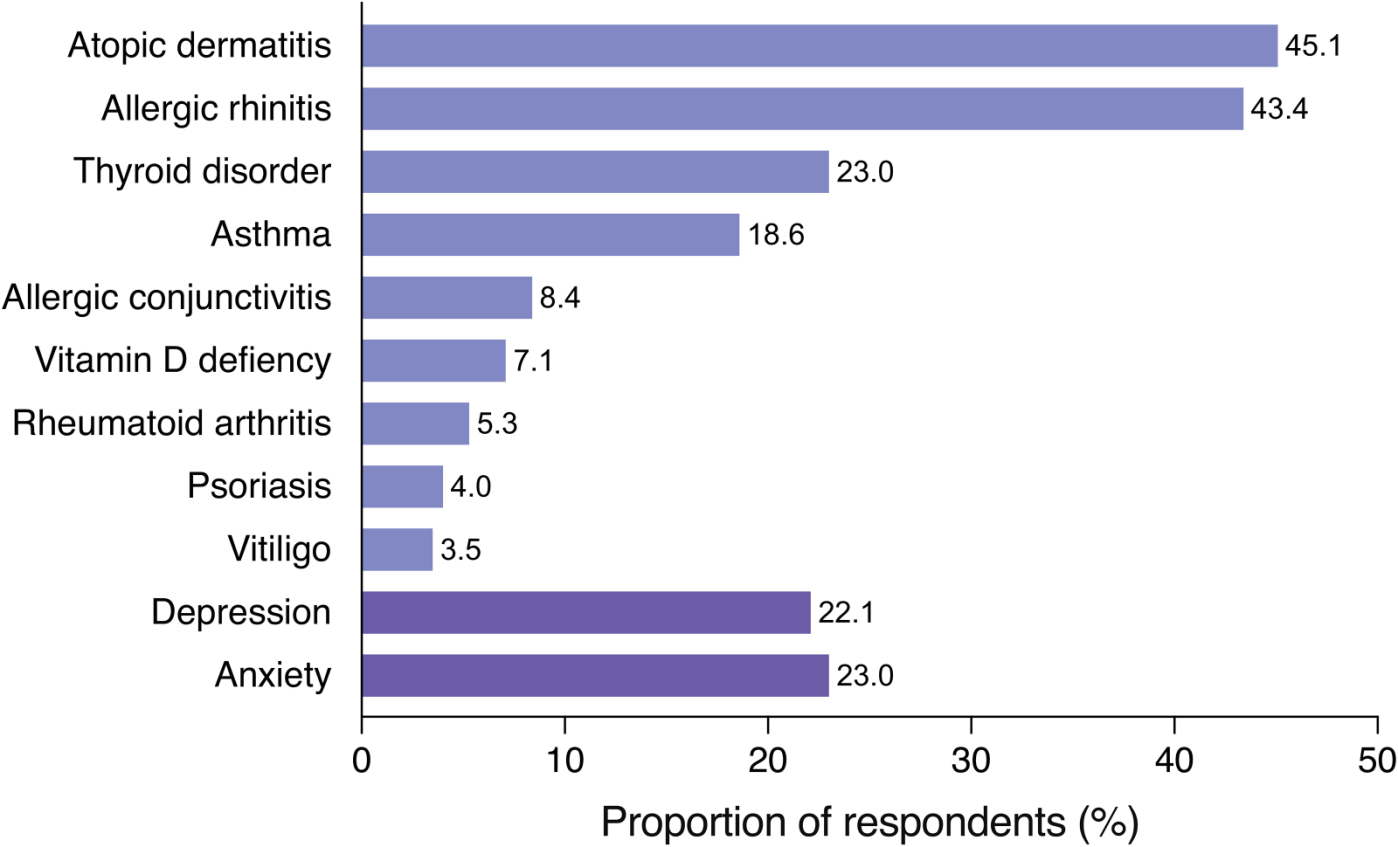
Comorbidities of individuals with alopecia areata (AA). The proportion of respondents who reported having a diagnosis of selected comorbidities (N = 226).

Most respondents (82.7%) reported having at least one diagnosed comorbid disease. The most frequent inflammatory comorbid diseases were atopic dermatitis (45.1%) and allergic rhinitis (43.4%) (Figure 1). One fifth (21.7%) of the respondents had both of these concomitant inflammatory diseases. The prevalence of atopic dermatitis and allergic rhinitis was not affected by the extent of hair loss (data not shown). Almost a third of the respondents (30.5%) reported having a psychiatric diagnosis of depression or anxiety. Depression was reported by 22.1% and anxiety by 23.0% (Figure 1), whereas 11.9% reported both. Importantly, psychiatric diagnoses were typically received after the diagnosis of AA. The extent of hair loss did not affect the rate of depression or anxiety (data not shown).

**Figure 2.**
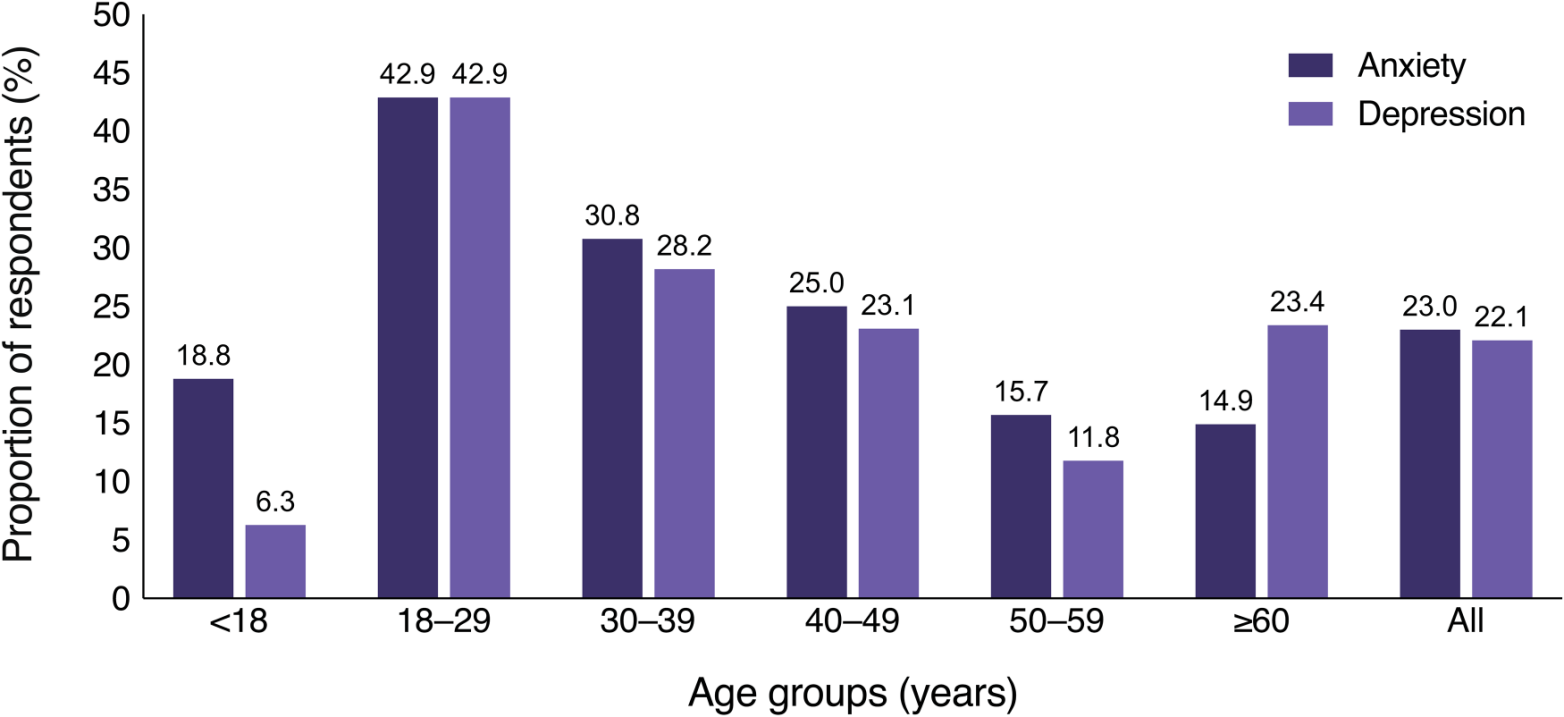
The proportion of anxiety and depression diagnoses by age group among individuals with alopecia areata (AA). The proportion of respondents by age group who reported having a diagnosis of anxiety or depression. A total of 226 responses were received, with 52 respondents reporting anxiety and 50 reporting depression.

The highest proportion of psychiatric diagnoses was found among young adults aged 18 to 29 (42.9% for both depression and anxiety). Anxiety was more prevalent than depression in respondents under 18 years of age (18.8% vs 6.3%), whereas depression was more prevalent among respondents that were 60 years or older (23.4% vs 14.9%) (Figure 2). Differences between the age groups were not assessed statistically.

### Negative Impact on Life

The impact of the AA on well-being was analyzed by dividing respondents into 2 groups based on the extent of hair loss: 46.5% of respondents had less than 50% hair loss, and 52.7% had at least 50% hair loss. Respondents with at least 50% hair loss were significantly less likely to report higher levels of overall well-being compared to those with less than 50% hair loss (OR, 0.60; 95% CI, 0.38-0.96; *P* = .03). In terms of work and functional ability, the difference between the groups was not statistically significant.

**Figure 3.**
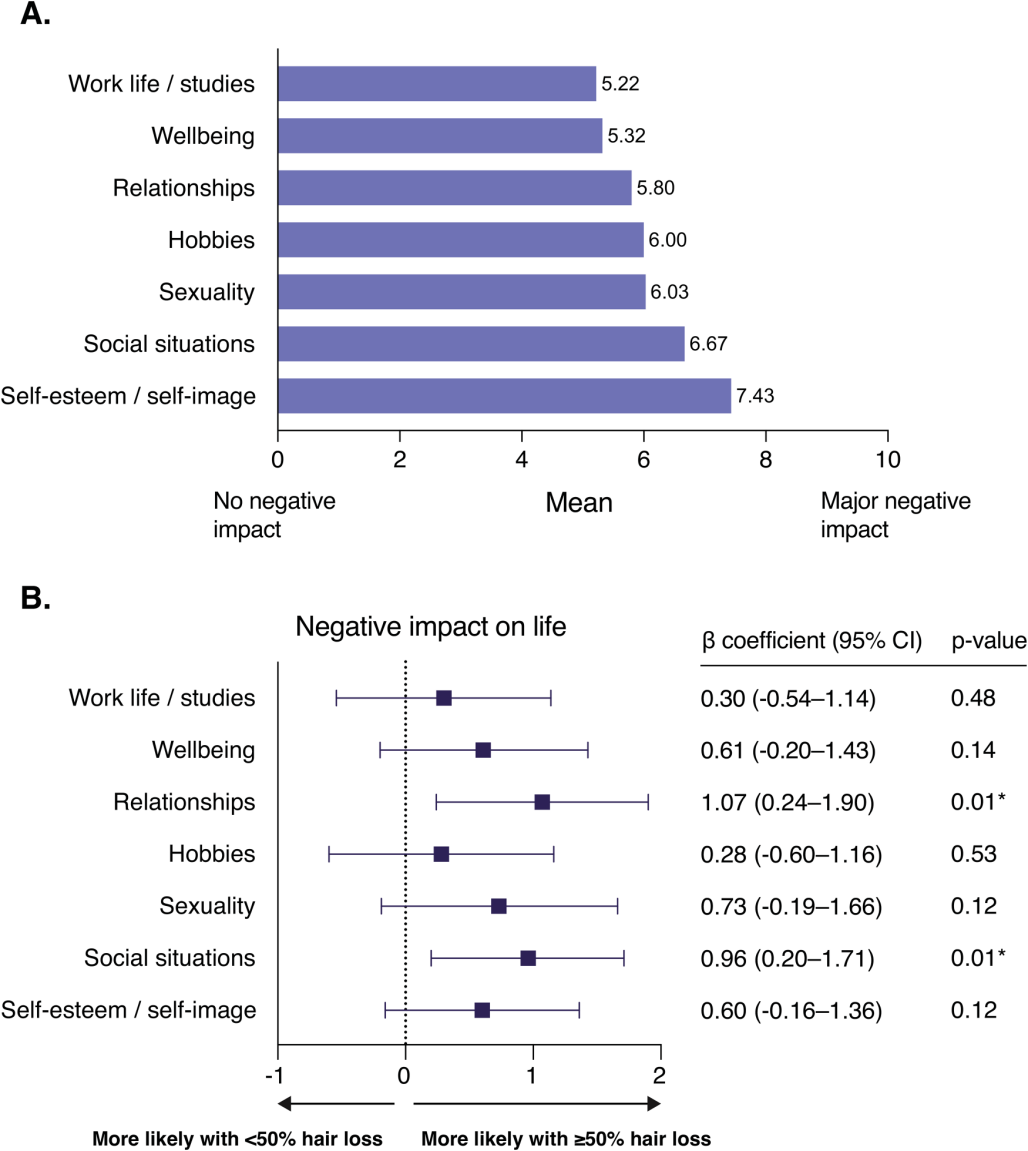
The negative impact of alopecia on different aspects of life. (A) The mean score of the responses regarding the negative impact of the disease on selected aspects of life. (B) Associations between the survey responses and the extent of hair loss (less than 50% or at least 50% hair loss) were analyzed by linear regression. I don’t know—responses were excluded from the analysis. Results are presented as linear regression coefficients (β) with 95% confidence intervals, and associated *P* values. *A *P* value below .05 was considered statistically significant.

When examining all respondents, the most significant negative impact on overall functioning was observed in the areas of self-esteem and self-image (Figure 3A). When examining the same areas in relation to hair loss, respondents with at least 50% hair loss were more likely to report higher levels of negative impact on relationships (β = 1.07; 95% CI, 0.24-1.90; *P* = .01) and social situations (β = 0.96; 95% CI, 0.20-1.71; *P* = .01), compared to those with less than 50% hair loss (Figure 3B). Additionally, respondents with at least 50% hair loss were more likely to agree that AA has made career life more challenging (OR, 1.66; 95% CI, 1.00-2.75; *P* = .05) and complicated relationships (OR, 1.67; 95% CI, 1.03-2.70; *P* = .04) compared to those with less than 50% hair loss.

### Treatment and Satisfaction to Treatment

The majority (70.8%) of respondents were not currently treated in any healthcare facility, 16.4% were treated in primary care, 4.9% in specialized care, and 4.4% in private clinics. Additionally, 82.7% of respondents reported that they had not used any treatment for AA in the past year. Among those who reported their treatment satisfaction, two-thirds (66.3%) were unsatisfied or very unsatisfied with their treatment, while 19.2% were moderately satisfied, and 14.5% were satisfied or very satisfied.

### Financial Burden

Respondents reported experiencing varying levels of financial concern related to healthcare fees, prescription medicines, and self-care products. Estimated AA-related costs in the previous year ranged from €0 to €7000, with an average annual cost of €843 and a median of €500. Over one-third (35.8%) reported spending €1000 or more on AA-related expenses in the past year.

The most common factor causing financial concerns was out-of-pocket expenses for wigs or permanent pigmentations. Altogether 62.8% of the respondents rated this to cause moderate or major financial concern. Majority of the respondents (79.2%) had either been granted aid for wig costs or were in the process of obtaining it. Only 5.3% of the respondents had been granted aid for permanent eyebrow pigmentation, while 9.7% had been denied aid for the same purpose.

The average annual aid reported for wigs was €606, with an additional average yearly out-of-pocket cost of €836. Furthermore, 60.6% of respondents stated that the aid received for wigs was insufficient to cover their medical or assessed treatment needs. Additionally, 30.1% of respondents reported having paid out-of-pocket for permanent pigmentation, with an average cost of €304.

### Sources of Information

When respondents were asked how often they use certain sources of information on a scale where 1 = never and 5 = very often, respondents most frequently mentioned social media (mean 3.55) and other AA patients (mean 3.42). In contrast, healthcare professionals were rarely mentioned as a source of information (mean 1.86). More than half of the respondents expressed a need for information about treatment options (59.7%), learning from other AA patients’ experiences (51.9%), and receiving advice on how to obtain state aid for wigs or eyebrow pigmentation (62.5%).

## Discussion

This survey highlights the significant burden of AA on various aspects of life for individuals with AA in Finland. Importantly, the study examined a wide range of themes—including mental and physical comorbidities, emotional and social well-being, access to treatment and support, financial impact, and sources of information—offering a comprehensive view of the lived experience with AA. This type of detailed, self-reported information is not available from health registries, underscoring the unique value of survey-based data in understanding the everyday challenges faced by individuals with chronic skin disease.

A majority of respondents reported at least one diagnosed comorbid disease, most frequently an inflammatory or psychiatric condition. Alopecia areata had a profound negative impact on self-esteem, self-image, and the ability to navigate social situations. Greater hair loss was associated with lower overall well-being and a more pronounced negative impact on daily life. In addition to the psychological burden, the financial strain of covering costs for wigs and pigmentation treatments further adds to the challenges faced by individuals with AA.

Notably, most respondents had not received any treatment in the past year, and among those who had, the majority were dissatisfied with their current treatment. This underscores the urgent need for more effective treatment options and support. Social media and peer support were the most common sources of information about AA for the respondents, highlighting a gap in patient education from healthcare professionals.

### Comorbidities and Mental Health Considerations

The most reported diagnosed autoimmune and inflammatory comorbidities among respondents were atopic dermatitis, allergic rhinitis, thyroid disease, and asthma. These findings align with previous registry-based studies,^6,7,9^ although our study found somewhat higher prevalence rates. This discrepancy may be due to selection bias, as individuals with multiple conditions and a higher disease burden may be more inclined to participate in surveys to share their experiences. Additionally, since the data are self-reported, there is a possibility of recall bias or misclassification compared to register-based diagnoses. Furthermore, the study design does not allow for conclusions about direct causality.

Chronic skin conditions are known to be associated with an increased risk for psychiatric disorders.^14^ In our survey, 23.0% of respondents reported a comorbid diagnosis of anxiety, and 22.1% reported depression. These findings are consistent with previous studies,^5,7,10^ which demonstrate that psychiatric comorbidities are more prevalent in individuals with AA compared to controls, with rates similar to those seen in other chronic skin conditions. The relapsing and unpredictable nature of AA, combined with its impact on appearance, may contribute to these mental health challenges.

Anxiety and depression were most frequently reported by young adults aged 18 to 29, a group already known to have a higher prevalence of these conditions in the Finnish general population.^15^ However, our findings suggest that the prevalence among young adults with AA may be even higher than in the general population, consistent with previous research.^16^ Younger individuals, particularly those with a recent diagnosis, may be more vulnerable to mental health issues compared to those who have lived with the condition for decades and have had more time to adapt. This may explain why older adults reported lower levels of anxiety and depression.

Importantly, our study highlights that while individuals with AA often receive support from peers, professional support remains limited. Even infrequent visits to healthcare professionals could provide valuable opportunities for assessing comorbidities such as depression and anxiety and offering guidance on available treatment options. Strengthening professional support may also help prevent or mitigate these psychological impacts.

### Impact of Hair Loss Severity on Well-Being

We examined the impact of self-reported hair loss on survey responses, using a 50% cutoff to define the extent of hair loss. In line with this, the Severity of Alopecia Tool measures scalp hair loss as a percentage, from 0% (no hair loss) to 100% (complete hair loss), with respondents experiencing 50% or more classified as having a severe condition.^17^ Based on this classification, we found that respondents with at least 50% hair loss were more likely to report poorer well-being and a negative impact on their careers and relationships, as well as experiencing greater difficulties in social situations, compared to respondents with less than 50% hair loss.

These findings suggest that individuals with greater hair loss experience the highest disease burden, aligning with previous findings,^8,18^ and may require prioritized healthcare resources. Currently, individuals with AA are rarely followed by healthcare professionals, yet those with extensive hair loss may benefit from more structured follow-up. Increased professional engagement could help in managing both the psychological and physical aspects of the disease while ensuring timely access to new treatment options.

### Financial Burden and Inequality in Access to Support

This survey highlights the significant financial burden of AA, with respondents reporting an average annual out-of-pocket cost of €836 for wigs and pigmentation treatments. This is slightly lower than the €1248 reported in German-speaking countries,^13^ likely reflecting differences in reimbursement policies and treatment availability.

In Finland, wigs and permanent pigmentation treatments are classified as medical rehabilitation aids, but amount of the payment commitment varies by region, creating inequalities in financial support. Often, coverage does not fully compensate for costs, leaving individuals to cover the remainder. Majority of the respondents expressed concerns about these disparities. These findings provide valuable insights for policymakers and patient organizations, offering updated data on the evolving costs of wigs and pigmentation treatments. This information is crucial for future discussions on healthcare reimbursement and support structures.

### Need for Improved Patient Support and Education

Survey respondents expressed a strong need for more information about their condition and treatment options, with social media and peer networks being their primary sources of information. In Finland, individuals with AA are rarely treated or followed by healthcare professionals, likely contributing to the lack of sufficient professional guidance. This is particularly relevant given that treatment options for AA have recently expanded, offering more opportunities for disease management.

Recent research into the pathophysiology of AA has identified JAK as key regulators of inflammation in hair follicles.^19,20^ Baricitinib (JAK1/2 inhibitor) has been approved for the treatment of severe AA in adults, while ritlecitinib (JAK3/TEC family kinase inhibitor) is approved for severe AA in both adults and adolescents. Both treatments are now available in Europe, the United States, and several other countries.^21^ However, access to these treatments varies, and their availability needs to be communicated effectively to individuals with AA. Increased engagement from healthcare professionals could help ensure that they receive up-to-date treatment information and necessary medical support.

## Limitations

Online surveys have various limitations that should be considered when interpreting the results. In this survey, respondents were recruited online through a patient organization, which may have introduced selection bias. However, as only half of the respondents were members of the organization, the survey also reached a broader population. Additionally, the study relied on self-reported diagnoses, hair loss percentages, and treatments received, which may lack the clinical accuracy of data retrieved from medical registers. This reliance on self-reported data introduces the possibility of recall bias or misclassification. Moreover, the descriptive nature of the data limits its ability to establish causal relationships or test specific hypotheses.

## Conclusion

This study highlights the broad range of challenges faced by individuals with AA. Alopecia areata is not merely a cosmetic condition but a chronic inflammatory disease associated with a high prevalence of both inflammatory and psychiatric comorbidities, significant psychological and economic burdens, and reduced quality of life. Individuals with AA rarely receive adequate follow-up in health care and report high levels of dissatisfaction with their current treatments. Moreover, there is a clear need for more reliable information about the disease, and individuals with AA may require additional support to manage the overall burden of the disease. Future research should also focus on treatment outcomes beyond hair regrowth, addressing the broader impacts of the AA on well-being.
